# Survive and thrive wellbeing programme: an innovative experiential wellbeing programme for trainees in Health Education England- Thames Valley (HEE-TV)

**DOI:** 10.1192/bjo.2021.447

**Published:** 2021-06-18

**Authors:** Haido Vlachos, Aisling Higham, Sara McDouall

**Affiliations:** 1 Health Education England Thames Valley; 2 Oxford University Hospitals NHS Foundation Trust, Heath Education England Thames Valley; 3 Royal Berkshire NHS Foundation Trust

## Abstract

**Aims:**

The aim of this project was to develop an experiential programme which encouraged trainees to develop their own processes for mental resilience acting to mitigate difficult work and life environments.

**Background:**

Doctors are at considerable risk of work-related stress, burnout and mental health problems, particularly trainees, many of whom are experiencing symptoms earlier in their career. The Thriving at Work Review, the British Medical Association and HEE all call for cultural and organizational change that works to prioritise, promote and enhance wellbeing by providing good working conditions and an atmosphere that encourages open discussion about mental health with access to appropriate support that destigmatises mental health.

**Method:**

Across HEE-TV we identified that there were no regular wellbeing initiatives for trainees, and specifically no psychologist-facilitated Cognitive Behavioural Therapy-style sessions to enhance resilience. Six schools identified a specific need for HETV-targeted resources focused on enhancing trainee mental wellbeing.

The current course has morning sessions that cover self-awareness, and afternoons are psychologist-facilitated sessions. The initial pilot was run for the School of Anaesthetics, and later offered to specialties with a General Medical Council-survey identified need. Multiple improved iterations of the course have been driven by detailed trainee feedback, including adding the psychology sessions to give trainees tools for self-help.

**Result:**

We triangulated feedback from attendees at the sessions, nominated trainee representatives from all specialties across Thames Valley via the Trainee Advisory Committee (TAC), and HEE-TV quality assessors. Feedback from trainees who attended was almost universally positive. The Quality Committee noted improvement of trainee morale in Anaesthetics and direct improvement in aspects of the learner environment that would not have happened without this intervention. The TAC endorsed this as one of the measures to support trainees in difficult learner environments. They also recommended it be rolled out for all as a preventative measure as there can be a time lag before items appear on the Risk Registers and are officially recognized as requiring support. The biggest measure of success is that HEE-TV have agreed to fund these sessions ongoing.

**Conclusion:**

We learned that an iterative response to trainee feedback and careful co-ordination is key to successful engagement via the training programme directors who arrange regional training programmes. This, and making the SAT course free at the point of use, makes it easier for trainees to access this programme. In addition we will be including the trainee voice is shaping bespoke aspects of the day for each specialty.

